# Custom RT-qPCR-array for glaucoma filtering surgery prognosis

**DOI:** 10.1371/journal.pone.0174559

**Published:** 2017-03-30

**Authors:** Iñaki Rodriguez-Agirretxe, Iker Garcia, Javier Soria, Tatiana Maria Suarez, Arantxa Acera

**Affiliations:** 1 Instituto Clínico Quirúrgico de Oftalmología, Bilbao, Spain; 2 Hospital Universitario Donostia, San Sebastian, Spain; 3 Bioftalmik Applied Research, Derio, Spain; Bascom Palmer Eye Institute, UNITED STATES

## Abstract

Excessive subconjunctival scarring is the main reason of failure of glaucoma filtration surgery. We analyzed conjunctival and systemic gene expression patterns after non penetrating deep sclerectomy (NPDS). To find expression patterns related to surgical failure and their correlation with the clinical outcomes. This study consisted of two consecutive stages. The first was a prospective analysis of wound-healing gene expression profile of six patients after NPDS. Conjunctival samples and peripheral blood samples were collected before and 15, 90,180, and 360 days after surgery. In the second stage, we conducted a retrospective analysis correlating the late conjunctival gene expression and the outcome of the NPDS for 11 patients. We developed a RT-qPCR Array for 88 key genes associated to wound healing. RT-qPCR Array analysis of conjunctiva samples showed statistically significant differences in 29/88 genes in the early stages after surgery, 20/88 genes between 90 and 180 days after surgery, and only 2/88 genes one year after surgery. In the blood samples, the most important changes occurred in 12/88 genes in the first 15 days after surgery. Correspondence analyses (COA) revealed significant differences between the expression of 20/88 genes in patients with surgical success and failure one year after surgery. Different expression patterns of mediators of the bleb wound healing were identified. Examination of such patterns might be used in surgery prognosis. RT-qPCR Array provides a powerful tool for investigation of differential gene expression wound healing after glaucoma surgery.

## Introduction

Glaucoma is characterized by progressive deterioration of retinal nerve fiber layer and optic nerve, leading to defects in the visual field and optic atrophy. In most cases, the condition is associated with increased intraocular pressure (IOP) caused by obstruction of drainage of aqueous humor from the eye. Currently, the main treatment for glaucoma is reduction of IOP using drugs and laser or filtration surgery.

Glaucoma Filtering Surgery (GFS) success depends primarily on the creation and maintenance of a communication channel between the anterior chamber and subconjunctival space. The conjunctival and episcleral healing is the main limiting factor in this surgery; postoperative IOP depends on the balance between scarring and tissue regeneration [1]. Thus, complete healing would cause failure of filtration surgery. In contrast, lack of healing would result in ocular hypotony.

Numerous pharmacological strategies have been employed to modulate conjunctival scarring. These include corticosteroids [2], antimitotics (5-fluorouracil, 5-FU and mitomycin C, MMC) [3–5], beta-radiation [6,7], inhibitors of growth factors (lederlimumab, bevacizumab) [8,9], and metalloproteinase inhibitors (ilomastat) [10]. However, some of these drugs are relatively nonspecific and lead to complications such as hypotony, endophthalmitis, etc.

Healing after glaucoma surgery consists of several stages: vascular response, coagulation, inflammation, proliferation and tissue remodeling [11]. Knowledge of the molecular mechanisms regulating this process should help in the development of new treatments for improved healing control. Likewise, categorization of patients based on gene expression profiles of the conjunctiva and Tenon’s capsule could help on identifying cases with high risk of failure, and thus improve the therapeutic options.

Reverse Transcription Real-Time Quantitative PCR Array (RT-qPCR Array) is the most reliable, specific and sensitive technology for analyzing the expression of a specific panel of genes. We present a customized RT-qPCR Array, used to analyze the expression of 88 genes implicated in the wound-healing process. These genes encode extracellular matrix (ECM) remodeling factors, inflammatory cytokines, as well as growth factors and main signaling molecules.

The purpose of this study was to investigate the conjunctival and systemic gene expression patterns at several different time points before and after non penetrating deep sclerctomy (NPDS), to correlate them with the clinical outcomes of the surgery, and to search for gene expression patterns related to surgical success and failure.

## Materials and methods

### Patients

The study used 11 eyes from 11 caucasian patients (four men and seven women, with a mean age ± standard deviation, SD of 67.16 ± 5.94 years) with uncontrolled primary open-angle glaucoma (POAG). The mean number (± SD) of preoperative topical anti-glaucoma drugs was 2.16 ± 0.75. Uncontrolled glaucoma was defined as an IOP over 21 mmHg after taking the maximum tolerated medication, with characteristic visual field and optic disc changes. Patients were recruited from the Glaucoma Unit of the Instituto Clínico Quirúrgico de Oftalmología (Bilbao, Spain), and Mendaro Hospital (Mendaro, Spain), after obtaining their signed informed consent.

The exclusion criteria were: concomitant administration of steroids or antimetabolites, previous anti-glaucoma surgery or conjunctival incisional surgery, cataract surgery during the three months before NPDS, diabetic retinopathy and other causes of ocular neovascularization. We also excluded cases of glaucoma with high risk of failure such as neovascular, aphakic, inflammatory, juvenile, traumatic, and postoperative glaucoma.

### Ethics statement

The study followed the principles established in the Declaration of Helsinki and was approved by the Euskadi Review Board (ethics committee for clinical research) of Cruces Hospital. Written informed consent was obtained from all the study participants and all samples were anonymized to preserve patient confidentiality.

### Study design

**Fig 1:**
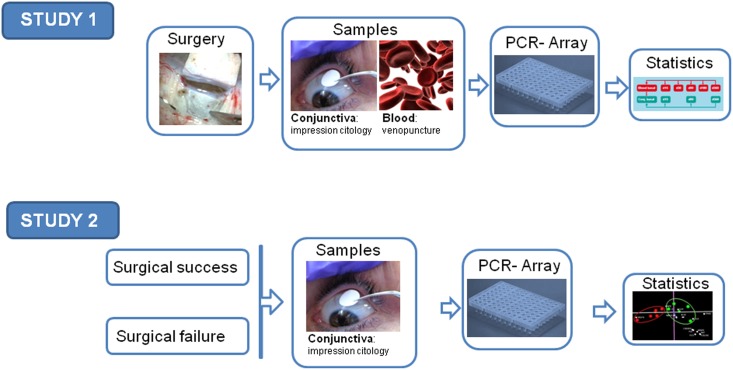
Workflow for PCR-Array analysis of glaucoma filtering surgery. Surgical failure was defined as an IOP ≥21 mmHg in the absence of ocular hypotensive treatment. In this study, we enrolled 11 patients (five patients with surgical failure and six patients from the first study).

The work was divided into two consecutive studies. In the first study, we performed a prospective analysis of the wound-healing gene expression profiles of six patients after NPDS. Conjunctival samples (obtained by impression cytology, IC) and peripheral blood samples were collected before the operation and 15, 90,180, and 360 days after surgery. We examined gene expression patterns at these time points. In the second study, we conducted a retrospective analysis correlating the late conjunctival gene expression (a year or more after the intervention), and the success or failure of the NPDS. From each patient, a single conjunctival sample was collected using IC. (Fig 1)

### Surgical technique and follow-up

NPDS was performed under topical anesthesia by the same surgeon (IRA). After excision of the deep scleral flap, a non-degradable HEMA implant (Esnoper, AJL Ophthalmic, Miñano, Álava, Spain) was sutured to the scleral bed with one 10–0 nylon suture. No antimetabolites were employed during the surgical procedure or in the follow-up. All patients received tobramycin and dexamethasone drops during one month after the surgery, in a decreasing pattern (Tobradex, AlconCusi, El Masnou, Barcelona, Spain). Follow-up consisted of biomicroscopic examination of anterior segment, checking bleb characteristics (morphology and presence of corkscrew vessels and epithelial microcysts), and IOP using Goldmann applanation tonometry.

### Sample collection

For IC, cellulose acetate membrane (HAWP304, Millipore, Bedford, MA, USA) was applied onto the upper bulbar conjunctiva after instillation of topical anesthetic (Colircusi anestésico doble, Alcon Cusi, El Masnou, Barcelona, Spain). IC samples were used to study preoperative ocular surface (PAS-hematoxylin staining) and for RNA purification to analyze gene expression profiles before and after surgery.

IC samples for PAS-hematoxylin staining were obtained on 5×5 mm strips of cellulose acetate and immediately fixed in 96% ethanol. IC samples for gene expression analysis were obtained from the upper bulbar conjunctiva at the surgical site, by applying both sides of an 8 mm-diameter disc. These discs were immediately placed in an RNA conserving buffer (RNAprotect Cell Reagent, Qiagen, Germantown, MD, USA) and stored at 4°C until use.

At the same time, peripheral blood samples were collected with standard methods using PAX gene blood collection tubes (Qiagen) for RNA stabilization.

### PAS-hematoxylin staining

PAS-hematoxylin staining was performed according to the Locquin and Langeron protocol modified by Rivas [12]. These samples were analyzed microscopically to establish the degree of metaplasia of the non-secreting cells, the presence of cytoplasmic or nuclear alterations, and cytoplasm-nucleus ratio.

### RT-qPCR array design

The search for genes related to the healing process was conducted in three stages. In the first stage, a subset of genes was selected through a literature review (PubMed). In the second stage, another subset of genes was obtained from the bioinformatic analysis of functional networks focused on wound healing and related scarring processes (http://geneontology.org/, DAVID, EGAN). In the third stage, 88 genes were selected after processing both subsets and discarding redundancies. Subsequently, a custom 96-well plate RT-qPCR Array containing the 88 selected genes was designed and produced specifically for this study (SA Biosciences, Frederick, MD, USA) (Table 1). The remaining wells corresponded to the five housekeeping genes, a negative genomic DNA control, and positive reverse transcription and PCR controls.

**Table 1:** List of genes selected and analyzed by RT-q PCR Array.

Gene Symbol	RefSeq (more than one if isoforms exist)	Cat#/PrimerID	RefSeq detected
*BAX*	NM_004324.3, NM_138761.3, NM_138763.3, NM_138764.4,	PPH00078B	NM_004324 and also NM_138765; NM_138764; NM_138763; NM_138761
*BCL2*	NM_000633.2, NM_000657.2	PPH00079B	NM_000633 and also NM_000657
*BTG2*	NM_006763.2	PPH01750B	NM_006763
*CASP1*	NM_001223.3, NM_033292.2, NM_033293.2, NM_033294.2, NM_033295.2	PPH00105B	NM_033292 and also NM_001223; NM_033295; NM_033294; NM_033293
*CASP3*	NM_004346, NM_032991	PPH00107B	NM_004346 and NM_032991
*CD44*	NM_000610.3, NM_001001389.1, NM_001001390.1, NM_001001391.1, NM_001001392.1	PPH00114A	NM_000610 and also NM_001001392; NM_001001391; NM_001001390; NM_001001389
*CDH1*	NM_004360	PPH00135E	NM_004360
*CDKN1A*	NM_078467, NM_000389	PPH00211E	NM_000389 and also NM_078467
*CDKN2A*	NM_000077.3, NM_058195.2, NM_058197.3	PPH00207B	NM_000077 and also NM_058197; NM_058195
*CEBPD*	NM_005195	PPH00776F	NM_005195
*CLU*	NM_203339, NM_001831	PPH00243E	NM_001831 and also NM_203339
*COL3A1*	NM_000090	PPH00439E	NM_000090
*CREBBP*	NM_004380, NM_001079846	PPH00324E	NM_004380 and also NM_001079846
*CTGF*	NM_001901	PPH00550F	NM_001901
*CTNNB1*	NM_001904, NM_001098209, NM_001098210	PPH00643E	NM_001904 and also NM_001098209; NM_001098210
*E2F1*	NM_005225	PPH00136F	NM_005225
*EGF*	NM_001963.3	PPH00137B	NM_001963
*EGFR*	NM_005228.3, NM_201282.1, NM_201283.1, NM_201284.1	PPH00138B	NM_005228 and also NM_201284; NM_201283; NM_201282
*ERBB2*	NM_001005862.1, NM_004448.2	PPH00209B	NM_004448 and also NM_001005862
*EREG*	NM_001432.2	PPH11303E	NM_001432
*ESR1*	NM_000125.3, NM_001122740.1, NM_001122741.1, NM_001122742.1	PPH01001A	NM_000125 and also NM_001122740; NM_001122741; NM_001122742
*F2*	NM_000506.3	PPH01158E	NM_000506
*FAS*	NM_000043.3, NM_152871.1, NM_152872.1, NM_152873.1, NM_152874.1, NM_152875.1, NM_152876.1, NM_152877.1	PPH00141B	NM_000043 and also NM_152877; NM_152876; NM_152875; NM_152874; NM_152873; NM_152872; NM_152871
*FGF2*	NM_002006	PPH00257C	NM_002006
*FLT1*	NM_002019	PPH00375C	NM_002019
*FN1*	NM_002026.2, NM_054034.2, NM_212474.1, NM_212475.1, NM_212476.1, NM_212478.1, NM_212482.1	PPH00143B	NM_002026 and also NM_054034; NM_212474; NM_212475; NM_212476; NM_212478; NM_212482
*HBEGF*	NM_001945.2	PPH02589A	NM_001945
*HGF*	NM_000601.4, NM_001010931.1, NM_001010932.1, NM_001010933.1, NM_001010934.1	PPH00163B	NM_000601 and also NM_001010932; NM_001010934; NM_001010933; NM_001010931
*HIF1A*	NM_001530.3, NM_181054.2	PPH01361B	NM_001530 and also NM_181054
*HSPD1*	NM_002156.4, NM_199440.1	PPH01205A	NM_002156 and also NM_199440
*HTT*	NM_002111.6	PPH05750E	NM_002111
*ICAM1*	NM_000201.2	PPH00640F	NM_000201
*IFNB1*	NM_002176.2	PPH00384E	NM_002176
*IFNG*	NM_000619.2	PPH00380B	NM_000619
*IL1A*	NM_000575.3	PPH00690A	NM_000575
*IL1B*	NM_000576.2	PPH00171B	NM_000576
*IL6*	NM_000600.3	PPH00560B	NM_000600
*IL8*	NM_000584.2	PPH00568A	NM_000584
*IL10*	NM_000572.2	PPH00572B	NM_000572
*IL18*	NM_001562.2	PPH00580B	NM_001562
*ITGA2B*	NM_000419.3	PPH00670A	NM_000419
*ITGB2*	NM_000211.3, NM_001127491.1	PPH00679E	NM_000211 and also NM_001127491
*ITGB3*	NM_000212.2	PPH00178C	NM_000212
*JUN*	NM_002228.3	PPH00095A	NM_002228
*JUNB*	NM_002229.2	PPH00179A	NM_002229
*MAPK1*	NM_002745.4, NM_138957.2	PPH00715B	NM_002745 and also NM_138957
*MET*	NM_000245.2, NM_001127500.1	PPH00194A	NM_000245 and also NM_001127500
*MIF*	NM_002415.1	PPH00548E	NM_002415 and also XM_002345496.1
*MLL2*	NM_003482.3	PPH18833A	NM_003482
*MMP1*	NM_002421.3, NM_001145938.1	PPH00120B	NM_002421 and NM_001145938.1
*MMP2*	NM_001127891.1, NM_004530.4	PPH00151B	NM_004530 and also NM_001127891
*MMP3*	NM_002422.3	PPH00235E	NM_002422
*MMP9*	NM_004994.2	PPH00152E	NM_004994
*MMP25*	NM_022468.4	PPH57607B	NM_022468
*MYC*	NM_002467.4	PPH00100A	NM_002467 and XM_001725281; XM_001725300
*NCOA6*	NM_014071.2	PPH05909A	NM_014071
*NF1*	NM_000267.2, NM_001042492.1, NM_001128147.1	PPH02089E	NM_000267 and also NM_001042492; NM_001128147
*NFKB1*	NM_003998.2	PPH00204E	NM_003998
*NGF*	NM_002506.2	PPH00205E	NM_002506
*PDGFA*	NM_002607.5, NM_033023.4	PPH00217B	NM_002607 and also NM_033023
*PDGFB*	NM_002608.2, NM_033016.2	PPH00488E	NM_002608 and also NM_033016
*PDGFRA*	NM_006206.4	PPH00219B	NM_006206
*PDGFRB*	NM_002609.3	PPH00477B	NM_002609
*PLAU*	NM_001145031.1, NM_002658.3	PPH00796B	NM_002658 and also NM_001145031
*PLG*	NM_000301.2	PPH02587E	NM_000301
*PPARA*	NM_005036.4, NM_001001928.2	PPH01281B	NM_005036 and also NM_001001928
*PTGS2*	NM_000963.2	PPH01136E	NM_000963
*PTK2*	NM_005607.3, NM_153831.2	PPH02827A	NM_005607 and also NM_153831
*RBPJ*	NM_005349.2, NM_203284.1, NM_203283.1, NM_015874.3	PPH06319E	NM_005349 and also NM_203283; NM_203284; NM_015874
*RELA*	NM_021975.3, NM_001145138.1	PPH01812B	NM_021975 and also NM_001145138
*SERPINE1*	NM_000602.2	PPH00215E	NM_000602
*SMARCA4*	NM_001128844.1, NM_001128845.1, NM_001128846.1, NM_001128847.1, NM_001128848.1, NM_001128849.1, NM_003072.3	PPH10099A	NM_003072 and also NM_001128844; NM_001128845; NM_001128846; NM_001128847; NM_001128848; NM_001128849
*SP1*	NM_003109.1, NM_138473.2	PPH01482A	NM_138473 and also NM_003109
*SPP1*	NM_001040058.1, NM_000582.2, NM_001040060.1	PPH00582E	NM_000582 and also NM_001040058; NM_001040060
*SRC*	NM_005417.3, NM_198291.1	PPH00103C	NM_005417 and also NM_198291
*STAT6*	NM_003153.3	PPH00760B	NM_003153
*TFAP2A*	NM_003220.2, NM_001032280.2, NM_001042425.1	PPH06072E	NM_003220 and also NM_001032280; NM_001042425
*TFF1*	NM_003225.2	PPH00998B	NM_003225
*TGFA*	NM_001099691.1, NM_003236.2	PPH00378A	NM_003236 and also NM_001099691
*TGFB1*	NM_000660.3	PPH00508A	NM_000660
*TGFB2*	NM_001135599.1, NM_003238.2	PPH00524B	NM_003238 and also NM_001135599
*TGFB3*	NM_003239.2	PPH00531E	NM_003239
*TNF*	NM_000594.2	PPH00341E	NM_000594
*TNFRSF1A*	NM_001065.2	PPH00346B	NM_001065
*TP53*	NM_000546.4, NM_001126112.1, NM_001126113.1, NM_001126114.1, NM_001126115.1, NM_001126116.1, NM_001126117.1	PPH00213E	NM_000546 and also NM_001126112; NM_001126113; NM_001126114; NM_001126115; NM_001126116; NM_001126117
*VCL*	NM_003373.3, NM_014000.2	PPH02077E	NM_003373 and also NM_014000
*VEGFA*	NM_001025366.1, NM_001025367.1, NM_001025368.1, NM_001025369.1, NM_001025370.1, NM_001033756.1, NM_003376.4	PPH00251B	NM_003376 and also NM_001025366; NM_001025367; NM_001025368; NM_001025369; NM_001025370; NM_001033756
*VTN*	NM_000638.3	PPH00253E	NM_000638
*ACTB*	NM_001101		
*GAPD*	NM_002046		
*HPRT1*	NM_000194		
*RPL13A*	NM_012423		
*B2M*	NM_004048.2		
*GDC*^a^			
*RTC*^b^			
*PPC*^c^			

^a^ Genomic DNA Control (GDC) primer set that specifically detects nontranscribed genomic DNA contamination with a high level of sensitivity.

^b^ Reverse Transcription Controls (RTC) to test the efficiency of the RT PCR Array.

^c^ Positive PCR Controls (PPC) to test the efficiency of the polymerase chain reaction itself using a pre-dispensed artificial DNA sequence and the primer set that detects it.

### RNA purification and RT-qPCR array processing

Total RNA was isolated from blood and conjunctiva. Whole peripheral blood was collected in PAXgene tubes (PreAnalytics) to grant RNA stabilization. After centrifugation, the supernatant was discarded and the pellet was treated with a series of buffers and silica-column purified following the manufacturer’s instructions (PAXgene Blood RNA kit, PreAnalytics, Qiagen). Thus, total blood RNA was purified for the study.

Conjunctival IC discs were processed with RNeasy Plus Micro Kit (Qiagen), with slight modifications of the manufacturer's protocol to perform an on-membrane lysis [13]. These modifications include a thorough vortexing during cell lysis to ensure maximum RNA recovery from both sides of the disc. Additionally, after transferring the cell lysate to the spin columns provided with the kit, the IC membrane alone was spun again to recover as much lysate as possible before this processing. Finally, after having added the elution buffer to the column, 10 minutes incubation was performed prior to RNA elution. After elution, the eluate loaded again into the column, allowing incubation for additional 10 minutes to maximize RNA concentration. These steps considerably increased RNA yield.

The quantification and evaluation of RNA quality were performed using an Agilent 2100 Bioanalyzer and RNA 6000 Pico Kit for low sample amounts (Agilent Technologies, Santa Clara, CA, USA). This method provides data on the integrity of isolated RNA (RNA Integrity Number; RIN), from which it extrapolates the RNA concentration. Conjunctiva and blood samples with RNA concentrations above 4 and 25 ng/μl, respectively, were processed; those with RIN less than 6 were discarded in both cases. cDNA was synthesized by reverse transcription of RNA using the RT^2^ First Strand Kit (SA Biosciences), following the manufacturer’s instructions. PCR was performed in iQ5 Thermal Cycler (Bio-Rad, Munich, Germany), using diluted cDNA as template (10 μl of cDNA, 10 μl of Genomic DNA Elimination Mixture and 91 μl of RNAse free water) and the RT2 SYBR Green qPCR Master Mix (SA Biosciences), according to the manufacturer's guidelines. All samples were analyzed in duplicate for each gene set.

### Generation of expression values

Quantification results were normalized against housekeeping genes (*ACTB*, *GAPDH*, *HPRT1*, *B2M* and *RPL13A*), whose expression is constitutive under our experimental conditions. To do that, we normalized the primary data, represented in threshold cycle (Ct), using the geometric mean of the Cts of the constitutive genes included in each plate. Then expression changes were calculated using the **Δ**Ct method, followed by the calculation of up or down regulation fold.

### Statistical analysis

According to the small sample size, nonparametric tests were used for statistical comparisons. The Wilcoxon test was used to compare both types of samples (blood and conjunctiva) with their baseline levels.

Correlation between gene expression and IOP was examined using Spearman’s correlation test. Gene expression patterns related to surgical success and failure were identified using Correspondence Analysis (COA). Statistical analysis was performed using SPSS 19.0 (SPSS Sciences, Chicago, IL, USA). The level of statistical significance was P <0.05.

## Results

None of the patients had intraoperative or postoperative complications. Regarding to the existence of systemic diseases, two patients were hypertensive patients treated with angiotensin-converting-enzyme inhibitors (ACE inhibitors), one patient suffered hypothyroidism treated by hormone replacement therapy and two patients were taking gastric protector. Finally, one patient was non-insulin dependent diabetic and was being treated with oral antidiabetic drugs.

**Fig 2:**
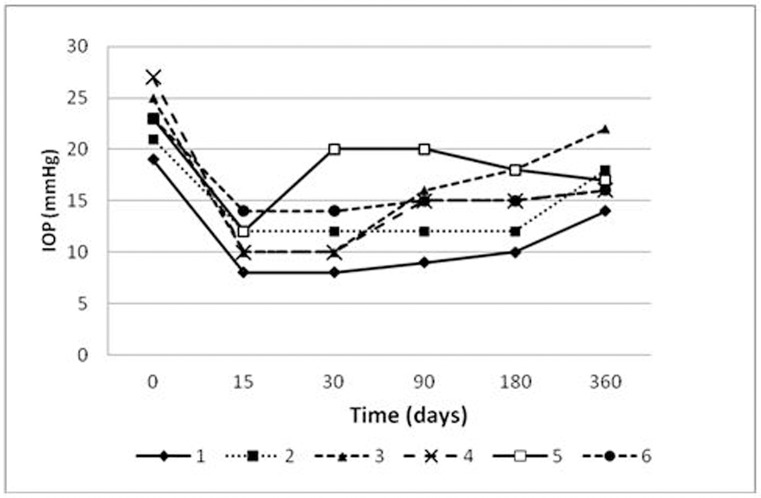
Schematic representation of the fluctuations in Intraocular Pressure (IOP) as a result of surgical treatment, at different stages of the study. In the group of patients analyzed in the first study, there was only one case of failure, a year after the surgery (IOP of 21 mmHg). For the remaining patients in this group, the intervention was successful, with IOPs maintained below 21 mmHg, which is the established criterion for determining the success of the surgery. The surgical failure case shows a scarring area where there it had been a channel allowing the drainage of aqueous humor.

IOP values decreased significantly in comparison with baseline values, without specifying pharmacological treatment in any of the cases (Fig 2). Surgery failed in one case, one year after the NPDS (patient 3 IOP: 22 mmHg), requiring reintroduction of drug therapy.

**Fig 3:**
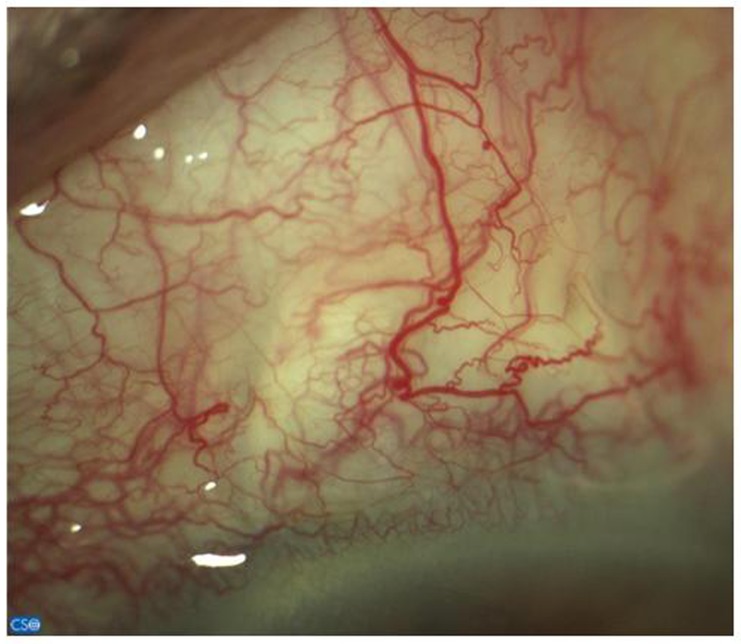
Bleb characteristics. Corkscrew vessels and microcysts.

Bleb morphology was predominantly diffuse (100% of patients after 15 days, decreasing to 60% of cases after one year). The remaining cases had flat blebs, except for patient 3 who presented a cystic bleb 180 days after the intervention. Corkscrew vessels were detected throughout the study, reaching its maximum 30 days after surgery (83% of patients) (Fig 3). In all cases, epithelial microcysts were found during the first 30 days after surgery, which later disappeared.

### PAS-hematoxylin staining of the conjunctiva

**Fig 4:**
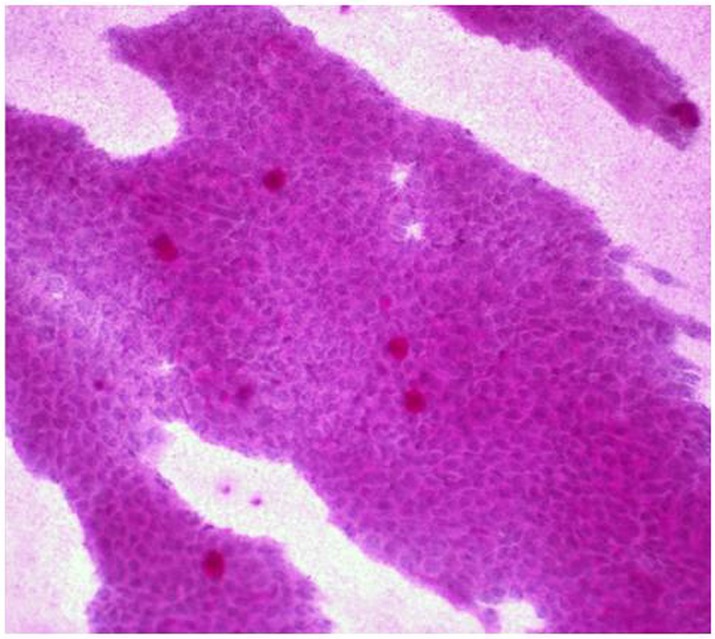
Microphotograph of preoperative conjunctival Impression Cytology (IC) with periodic Acid-Schiff-hematoxylin staining, obtained from a glaucoma patient. The nonsecretory epithelial cells are small and round and retain the intercellular junctions. The nuclei are large and N:C ratio is 1:2. The goblet cells are abundant, plump, and oval. Magnification 40x.

All patients included in the study had grade-0 squamous metaplasia in the preoperative conjunctiva (Fig 4). The epithelial cells were small and round, with eosinophilic cytoplasm. The nuclei were large, basophilic, with a nucleo-cytoplasmic ratio of 1:2. Goblet cells were abundant, plump, and oval and had an intensely PAS-positive cytoplasm. No deterioration of the ocular surface due to drug treatment was observed before the operation.

### Gene expression analyses

In the first study, where we compared conjunctival gene expression with baseline expression at each time point (prospective study) (S1 Table), we observed statistically significant changes for 29 of the 88 genes studied (Table 2). In the postoperative phase (15 days), were detected the most significant changes in gene expression (upregulation of *CDKN1A* and *CDKN2A*, *IL8*, *TGFA*, and *VEGFA* genes) were detected. In the second phase (90 days), we found expression changes in 20 genes (all of these were downregulated with the exception of *TGFA*). After one year, only two genes showed expression changes (downregulation of *TGFB1* and *ITGB3*).

**Table 2:** List of conjunctival genes showing expression differences at 15, 90, and 360 days after surgery.

Gene	Name	*P*	Fold	Days
*IL8*	Interleukin 8	0.017	140.57	0 to 15
*VEGF-A*	Vascular Endothelial Growth Factor alpha	0.033	7.01	
*CDKN 2A*	Cyclin-dependent kinase inhibitor 2A	0.017	5.96	
*CDKN 1A*	Cyclin-dependent kinase inhibitor 1A	0.017	4.08	
*TGF-A*	Transforming Growth Factor alpha	0.017	3.08	
*IL18*	Interleukin 18	0.017	-4.00	
*MYC*	Myelocytomatosis oncogene	0.017	-9.09	
*TGF-A*	Transforming Growth Factor alpha	0.042	2.17	0 to 90
*JUNB*	Jun B proto-oncogene	0.042	-2.00	
*MIF*	Macrophage migration Inhibitory Factor	0.024	-3.12	
*TGF B1*	Transforming Growth Factor beta 1	0.012	-3.33	
*HIF 1A*	Hypoxia-Inducible Factor 1 alpha	0.012	-3.57	
*SMARC A4*	SWI/SNF related, Matrix associated, Actin dependent Regulator of Chromatin, subfamily A, member 4	0.012	-3.57	
*CLU*	Clusterin	0.042	-3.84	
*IL 18*	Interleukin 18	0.006	-3.84	
*CD44*	Glycoprotein CD44	0.042	-4.00	
*TFAP 2α*	Transcription Factor AP-2 alpha	0.012	-4.54	
*TGF B3*	Transforming Growth Factor beta 3	0.024	-4.76	
*HTT*	Huntingtin	0.012	-5.55	
*EGFR*	Epidermal Growth Factor Receptor	0.006	-7.14	
*PDGF A*	Platelet Derived Growth Factor A	0.006	-8.33	
*BCL2*	B-Cell Lymphoma 2	0.024	-10.00	
*MLL2*	Myeloid Lymphoid Leukemia 2	0.012	-11.11	
*CTGF*	Conective Tissue Growth Factor	0.012	-14.28	
*MYC*	Myelocytomatosis oncogene	0.006	-16.66	
*TGF B2*	Transforming Growth Factor beta 2	0.006	-20.00	
*MMP2*	Matrix Metalloproteinase 2	0.024	-25.00	
*ITG B3*	Integrin beta 3	0.033	-1.06	0 to 360
*TGF B1*	Transforming Growth Factor beta 1	0.017	-4.16	

The analysis of gene expression changes in peripheral blood, relative to baseline (S2 Table), showed expression changes of 12 genes among the 88 genes studied (Table 3).

**Table 3:** List of genes in blood samples showing expression differences at 15, 90, and 360 days after surgery.

Gene	Name	*P*	Fold	Days
*IL 1β*	Interleukin 1	0.03	2.05	0 to 15
*CEBPD*	CCAAT / Enhancer Binding Protein Delta	0.009	1.7	
*HIF 1A*	Hypoxia-Inducible Factor 1 alpha	0.009	1.69	
*ITG β2*	Integrin beta 2	0.009	1.49	
*TNFRSF 1A*	Tumor Necrosis Factor Receptor Superfamily member 1A	0.017	1.29	
*CASP 1*	Caspase 1	0.003	1.12	
*MET*	Met proto-oncogene (hepatocyte growth factor	0.026	-1,96	0 to 90
*TGF B2*	Transforming Growth Factor beta 2	0.026	-2,7	
*HIF 1A*	Hypoxia-Inducible Factor 1	0.038	1.94	0 to 180
*EREG*	Epiregulin	0.038	1.97	
*IL 1β*	Interleukin 1	0.038	2.27	

These changes were more pronounced in the first 15 days (upregulation of *IL1B*, *CEBPD*, *HIF1A*, *ITGB2*, *TNFRSF1A* and *CASP1*). The remaining expression changes happened between 90, and 180 days after surgery. One year after the intervention (as in the conjunctiva), the baseline expression levels of most genes were restored, with the exception of *HIF1A*, *EREG* and *IL1B*.

### Correlation between gene expression and IOP

We found a significant correlation between gene expression and the values of IOP for 15 genes in the conjunctiva and only a single gene in the peripheral blood (Table 4) samples. Conjunctival *TGFA* and *IL18* showed the strongest correlation values.

**Table 4:** Correlation IOP-gene expression in blood and conjunctiva samples.

	Spearman coefficient r_s_	*P*
**Blood genes**		
*IL 1β*	-0.387	0.038
**Conjunctival genes**		
*IL 18*	0.604	0.008
*BCL2*	0.576	0.012
*PDGF A*	0.570	0.014
*TGF B3*	0.560	0.016
*CTGF*	0.547	0.019
*MMP2*	0.547	0.019
*MYC*	0.547	0.019
*COL3A1*	0.529	0.024
*CLU*	0.504	0.033
*TGF B2*	0.481	0.043
*MIF*	0.479	0.044
*TGF-A*	-0.668	0.002
*CDKN 2A*	-0.509	0.031
*CDKN 1A*	-0.499	0.035
*CASP 3*	-0.485	0.041

### Clustering gene expression patterns related to surgical success and failure

**Fig 5:**
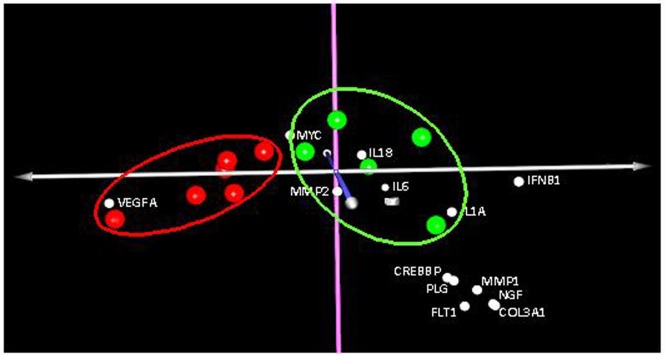
Correspondence Analysis (COA). Distribution of genes that separate the group of patients with successful surgery (green dots) from the patients with surgical failure (red dots).

Correspondence Analysis (COA) of gene expression of conjunctival samples in study 2 showed a clear clustering of patients with surgical success and failure (Fig 5). The statistical analysis revealed 20 genes whose expression differed significantly between these two groups (Table 5). From this analysis a clear over expression of *VEGFA* gene was related with failure of surgery whereas a notable decrease in *IFNB1* gene was also related.

**Table 5:** Genes with significant expression changes in patients with surgical failure group in comparison with those of the success group.

Gene	Fold	*P*
*VEGF-A*	3.00	0.0003
*HIF 1A*	-1.52	0.035
*MIF*	-1.62	0.025
*ITG B3*	-2.30	0.037
*MYC*	-2.55	0.017
*IL18*	-2.70	0.009
*CTGF*	-3.08	0.043
*MMP2*	-3.24	0.012
*BTG2*	-3.35	0.045
*CREBBP*	-3.65	0.044
*MMP3*	-4.90	0.040
*PLG*	-6.16	0.038
*FLT1*	-6.32	0.044
*MMP1*	-7.14	0.044
*NGF*	-7.65	0.049
*IL1A*	-7.69	0.015
*COL3A1*	-8.37	0.048
*F2*	-8.37	0.048
*IL6*	-8.84	0.038
*IFN β1*	-10.72	0.043

## Discussion

Despite the downward trend in glaucoma surgery since the nineteen-nineties [14], it is still a procedure with major advantages; it is inexpensive [15] and allows an IOP reduction more drastic [16] and less fluctuating than pharmacological treatment [17]. Although the most common glaucoma surgical technique worldwide is the trabeculectomy, some alternative interventions have been proposed to reduce the IOP. The NPDS could be considered as a variation of the trabeculectomy which does not require iridectomy and allows the aqueous humor to drain gradually, reducing IOP in a less abrupt manner. Long-term results seem to be not worse than those of trabeculectomy [18]. However, subconjunctival and episcleral scarring remain the main impediment to the long-term success of any filtering surgery [19], irrespective of whether it is perforating or non-perforating procedure [20].

Analysis of biochemical mechanisms involved in wound healing and failure of filtering surgery requires sampling of the conjunctiva and Tenon’s capsule. IC allows the collection of cells from the conjunctiva in a nearly painless and non-invasive way, for the purpose of analyses (cytology, flow cytometry, PCR…) and diagnosis. There are other different methods to obtain conjunctival cells such as brushing or biopsy, however these techniques are more invasive than IC and are not indicated in studies requiring repeated conjunctival sampling. Although collecting conjunctival cells from IC is technically challenging, the IC has been standardized and widely used in many studies for years [21–27]. Recently, Lopez-Miguel A et al., have published a paper which shows that the impression cytology is a robust and reproducible technique to obtain acceptable quantities of RNA [21]. In their work they conclude that there are no statistical differences between two methods employed for obtaining the sample: the conventional IC and the EyePrim device (OPIA Technologies, Paris, France). Previous studies from our research group were used to optimize the storage and processing conditions of the conjunctival IC samples and to provide reliable amounts of medium-high RNA quality, adequate for gene expression analysis [13,28,29]. In summary, IC is the technique that assures us the highest reproducibility and lower discomfort for the patient.

It could be argued that, using our technique, we only analyze the conjunctival epithelium, neglecting deeper strata such as conjunctival stroma and Tenon's capsule, associated with surgical failure [1]. However, firstly given the close relationship between the conjunctiva and anterior Tenon's capsule [30], the postoperative gene expression in these tissues should not differ substantially. Secondly, the low cellularity of Tenon’s capsule makes it difficult to extract its genetic material; in practice, it is usually extracted together with the conjunctiva. Thirdly, the typical location of the fibrosis under the conjunctival flap border in the case of surgical bleb revision could indicate the contribution of the conjunctiva to the fibrosis in a sort of epithelial mesenchymal transition. Unfortunately, this fact has not been demonstrated yet in glaucoma surgery. Moreover, not only conjunctival cells but also inflammatory cells could contribute to the surgical failure. In this respect it is worth mentioning that some changes could be due to the effect of the topical antiglaucoma drugs in the ocular surface. In the current clinical practice, we mainly operate patients with uncontrolled IOP in spite of the maximum tolerated medication. So, although these changes could be different among patients, they are unavoidable and should not interfere in the study; at last, they are expected to cause the same gene expression variations throughout different patients and sample collections, which would not affect our differential expression study.

To obtain a homogenous set of samples, we only enrolled patients with POAG. We excluded patients with glaucoma with high risk of surgical failure and those using supplementary antimetabolites, to rule out interference in the healing process. Furthermore, although the patients had received multiple topical treatments, which have been previously associated with surgical failure [31], they did not show significant changes in the ocular surface (as confirmed by the results of conjunctival impression cytology stained with PAS-hematoxylin).

In our knowledge, this is the first study in gene expression in humans after NPDS. Recently, Mahale A et al. have studied the fibrosis related gene expression after Ahmed glaucoma valve [32]. As in our study they have employed a fibrosis targeted PCR Array, however they have focused their study in a single-time point (median time about 25 months) which could be considered as a late capsule excision time.

Our results showed that, in the first stage, the main changes in conjunctival gene expression occurred on day 15 after the surgical trauma. In this phase, we observed three patterns of gene expression: upregulation (*CDKN1A* and *CDKN2A* genes, *IL8*, *TGFA*, and *VEGFA*), downregulation (*IL18* and *MYC*) and no change (other genes included in the study). The upregulated genes encode factors favoring chemotaxis of inflammatory cells (*IL8*) [33,34], angiogenesis (*VEGFA*, *IL8*) [35], and proliferation and migration of keratinocytes (*IL8* and *TGFA*) [36, 37], all of which are associated to processes typical of the first stage of wound healing [11]. Elevated expression of *IL8* was found in three patients and could be explained as a typical chemokine response in the acute phase of healing [37]. There are some doubts about the role of *CDKN* genes, which slow down the cell cycle. However, the overall effect of these genes on the cell cycle also depends on their effectors, which were not analyzed in this work. A decrease in the expression of *IL18* might be mediated by overexpression of *TGFA*, as it has been previously described [38]. It has been postulated that *IL18* exerts its proinflammatory effect in early stages of healing and shows expression changes at this stage [38]. The downregulation of *MYC* would have pro-healing effect, given the inhibitory effect of this protein on the process of wound healing [39].

In the intermediate phase of the healing process (90 days), we detected downregulation of some genes involved in crucial biological processes associated with healing. These genes have been implicated in the positive regulation of cell proliferation (*HIF1A*, *EGFR*, *MYC*, *MLL2*, and *TFAP2A*) [40, 41], response to wound healing (*CLU*, *CTGF*, *TGFB1*, *TGFB2*, *BCL2*, *PDGFA* and *MIF*) [42–44], fibroblast migration (*TGFB1*, *TGFB2*, *TGFB3*, *MMP2* and *CTGF*) [45–48] and angiogenesis (*PDGF*) [49–51].

In the final stage of the first study, one year after the surgery, virtually no changes in conjunctival gene expression were observed and basal expression levels were recovered. Only *TGFB1* and *ITGB3*, involved in the activation of healing showed a decrease in their expression [52].

Gene expression analysis in blood samples found the most significant changes during the first 15 days after surgery. During this phase, genes associated with pro-inflammatory processes (*CASP1*, *IL1B*) [53], pro-apoptotic genes (*HIF1A* and *ITGB2*) [54] and in addition, genes associated with anti-apoptotic or anti-inflammatory processes such as *CEBPD* [55]. During the next stages, expression changes were minimal; some cell migration- and inflammation-related genes showed expression changes. It is clear that, despite detectable changes in overall gene expression, they are less pronounced in the blood and differ from those found in the conjunctiva. This finding, also reported by other authors [56], reflects the primarily local nature of the healing process. Unfortunately, we could not perform paired comparisons of gene expression between blood and conjunctiva throughout the survey, due to the small number of adequate conjunctival RNA samples.

Regarding the analysis of the correlation between IOP and gene expression, we found that the most significant changes occurred in two genes expressed in conjunctiva (*IL18* and *TGFA*). Whereas *IL18* gene showed a positive correlation, *TGFA* gene showed a negative correlation with IOP. This data together with the gene expression analysis suggest that local factors play an important role in the surgical outcome.

In our second study, we decided to analyze only conjunctival samples from patients undergoing glaucoma filtering surgery once the wound healing process had concluded. At this time, the first group (designated "success”) maintained IOP below 21 mmHg as a result of good filtration of aqueous humor. However, in the second group (called "failure"), the surgery had failed, thus being IOP levels over 21 mmHg. The comparison of gene expression levels performed using COA revealed on the one hand, a trend of association among patients with successful surgery and, on the other hand, among patients with failed surgery (Fig 5). This indicates that the expression of genes related to wound healing in patients with failed surgery, differs from that of patients for whom the treatment succeeded. As in the first study, we showed that the expression levels one year after intervention were comparable with the basal levels. Ultimately this analysis could theoretically differentiate between success and failure. From the genes with different expression levels in success group in comparison with failure group, *VEGFA* was the furthest gene from the origin of the graph and the closest to the failure group, thus contributing significantly to the differentiation of the latter group (Fig 5). This was consistent with the data shown in Table 5, which showed 3-fold higher expression of this gene in the failure group than in the success group. In the latter group, genes that contributed most to the separation were *IFNB1*, I*L1A*, *IL18*, *IL6*, *F2*, *COL3A1*, *NGF*, *MMP1*, *FTL1*, *PLG*, *MMP3 and CREBBP*, whose decreased expression hindered the success of glaucoma surgery.

On the assumption that gene expression levels one year after the NPDS would not differ from the preoperative status, our analysis could provide a predictive classifying tool for forecasting the success or failure of glaucoma operation. Prediction of the risk of scarring based on individual gene expression profiles has a great potential in the development of personalized and stratified therapies to prevent ocular fibrosis. The combination of therapeutic targets in different biological pathways could offer a more effective treatment than conventional monotherapies. In addition, patient management before, during and after the intervention (use of antimitotics, dosage, etc.) could be substantially improved with the use of such tools.

To our knowledge, this study is the first in which expression of a broad spectrum of genes after NPDS in patients with POAG has been examined. This RT-qPCR Array analysis allows to determine not only the roles and mechanisms of various mediators of the conjunctival wound healing process, but also to identify the mediators implicated in the prognosis of the surgery.

However, there are limitations in our study. First, due to the low number of patients, our results should be interpreted with caution until further studies with larger sample sizes will be carried out. Second, a subsequent validation study performed at a protein level would be desirable in order to confirm the gene expression levels determined. Unfortunately, due to the small number of adequate conjunctival RNA samples, we could not perform this kind of study. Regardless of these limitations, our findings demonstrate clear alterations in fibrosis related gene pathways in NPDS and these preliminary findings could help form the basis for systematic future investigations.

In conclusion, this study identified an expression profile of genes related to inflammation, angiogenesis and cell proliferation, whose expression levels change after glaucoma filtering surgery. This type of analysis might become a valuable predictive tool for forecasting the success or failure of glaucoma surgery.

## Supporting information

S1 Table The mean expression values of the genes analyzed in conjunctiva samples. (XLSX)

S2 Table The mean expression values of the genes analyzed in the blood samples. (XLSX)
